# The Utility of Routine Postoperative Radiographs Following Surgical Treatment of Traumatic Cervical Spine Injuries

**DOI:** 10.3390/jcm15062231

**Published:** 2026-03-15

**Authors:** Hershil Patel, Sapan Patel, Rohan I. Suresh, Vishal A. Khatri, Keerthana Srinivasan, Husni Alasadi, Evan Honig, Ryan Curto, Usman Zareef, Robin Fencel, Alexander Padovano, Louis J. Bivona, Daniel L. Cavanaugh, Eugene Y. Koh, Steven C. Ludwig, Julio J. Jauregui

**Affiliations:** Department of Orthopaedics, Division of Spine Surgery, University of Maryland School of Medicine, Baltimore, MD 21201, USA

**Keywords:** postoperative radiograph, cervical spine trauma, revision surgery, instrumentation failure, clinical utility, anterior cervical discectomy and fusion, posterior cervical spine fusion

## Abstract

**Background/Objectives**: Postoperative cervical spine radiographs are routinely obtained during in-hospital and follow-up period. We aim to evaluate the utility of postoperative radiographs for identifying instrumentation failure and the subsequent need for revision surgery in patients with traumatic cervical spine injuries. **Materials and Methods**: A retrospective chart review of patients who had surgical treatment for traumatic cervical spine injury was conducted. Clinical notes and radiographic reports were evaluated. Postoperative radiographs were obtained prior to discharge from the hospital, and subsequently at 2, 6, 12, 24 weeks, and 1 year. Patients who underwent revision surgery, described as any reoperation, were identified. The patients’ indications for surgery were evaluated. The results of postoperative radiographs that prompted a change in management and reoperation were analyzed. Sensitivity and specificity for postoperative radiographs were calculated. **Results**: A total of 295 patients were reviewed. The rate of revision surgery was 3.7% (*n* = 11). All 11 patients presented changes in clinical findings and physical exam, but only 3 patients (1%) were identified to have undergone revision surgery due to instrumentation failure seen on radiographs at 13, 89, and 112 days postoperatively, and none within the inpatient period. Two patients underwent revision surgery due to epidural hematoma, and six patients due to wound infection. The overall sensitivity and specificity of routine postoperative radiographs were 27% and 100%, respectively. **Conclusions**: Postoperative radiographs after cervical spine trauma have low clinical utility for predicting instrumentation failure in the absence of clinical findings, particularly in the inpatient period.

## 1. Introduction

Traumatic cervical spine injury represents a minority of blunt trauma presentations, but it remains one of the most clinically consequential injury patterns because missed or unstable lesions may result in substantial neurologic morbidity, long-term disability, and death [1,2,3]. Prior epidemiologic studies have estimated that cervical spine injury occurs in approximately 3.7% of trauma patients overall, with lower prevalence in alert patients (2.8%) and substantially higher prevalence in clinically unevaluable patients (7.7%); notably, nearly 41.9% of identified injuries are considered unstable [1]. Population-based data further demonstrate that traumatic cervical spine fractures occur at an incidence of 11.8 per 100,000 person-years, show a male predominance, and are most commonly associated with falls and motor vehicle collisions [4,5]. Because cervical trauma spans a broad range of severity, from stable fractures to osteoligamentous instability with spinal cord injury, its emergent evaluation relies on standardized clinical and radiographic protocols to identify injury, guide treatment, and estimate prognosis [2,3].

Given the potentially serious consequences of acute loss of reduction, hardware failure, graft-related complications, or missed postoperative deterioration, radiographs are routinely obtained after surgical fixation of cervical spine injuries [2,6]. The rationale for routine imaging varies, but it is commonly used to document alignment, assess construct integrity, establish a postoperative baseline, detect instrumentation-related complications even in asymptomatic patients, and provide medicolegal documentation [7,8]. However, because acute postoperative failure is relatively uncommon and many patients already have satisfactory intraoperative fluoroscopic assessment, the utility of routine postoperative imaging in the absence of new clinical or neurologic findings has increasingly been questioned. In a large cervical fusion series, Shau et al. found that abnormal radiographs alone changed treatment in only 6 of 972 postoperative clinic encounters, with an overall positive predictive value of 19.0% and negative predictive value of 93.9% [8]. Similarly, Grimm et al. reported that among patients with a normal history and physical examination, further clinical action was taken at only 5 of 879 visits (0.57%), whereas abnormal history and examination findings were far more likely to identify patients who ultimately required additional intervention [9]. In a trauma-specific thoracolumbar cohort, Pyun et al. found an in-hospital revision rate of only 1.3% (6/463), with worsening neurologic status driving reoperation more often than routine postoperative radiographs alone; notably, the sensitivity of routine inpatient radiographs was only 33.3% [10]. Together, these studies suggest that reoperation is more often driven by evolving symptoms or examination changes than by surveillance radiographs alone, particularly during the early postoperative period.

At the same time, not all postoperative imaging is equivalent. Selected advanced imaging may still play an important role when the clinical course is concerning or when standard radiographs are equivocal. For example, postoperative CT can better characterize hardware integrity, alignment, or nonunion in select cases, whereas postoperative MRI may be useful when there is concern for neurologic compression or hematoma rather than as indiscriminate routine surveillance [11,12]. Despite this broader imaging landscape, evidence specifically evaluating the utility of routine postoperative upright radiographs after surgical fixation for traumatic cervical spine injury remains limited. Therefore, the purpose of this study was to assess the utility of routine postoperative radiographs in cervical spine trauma patients following surgical fixation in order to predict the need for revision surgery, particularly in the inpatient period prior to discharge. We hypothesized that routine postoperative radiographs would have minimal clinical utility in guiding postoperative management and reoperation in the absence of new clinical findings, especially during the inpatient period.

## 2. Materials and Methods

### 2.1. Study Population

After obtaining Institutional Review Board (IRB) approval, retrospective cohort analysis was performed on patients who underwent operative treatment for traumatic cervical spine injuries over an eight-year period between January 2008 and December 2016 at a level I trauma center and tertiary referral academic institution. Patients were identified by CPT and ICD-9 codes from a physician-driven surgical database. CPT codes for posterior cervical arthrodesis (22590, 22595, 22600), anterior cervical arthrodesis (22551, 22554), and open treatment of cervical fracture (22319, 22326) were cross-referenced with ICD-9 codes for cervical fracture (805.00–805.08).

Patients were included if they (1) were at least fused level 1 due to trauma and (2) were at minimum 18 years of age. All patients had intraoperative fluoroscopy demonstrating adequate alignment of instrumentation. Prior to discharge, postoperative upright anteroposterior (AP) and lateral radiographs were obtained and reviewed by one of four spine surgeons and a radiologist as per standard protocol. Advanced imaging, including CT and MRI, was not obtained routinely for all patients as part of the standard postoperative surveillance pathway. Rather, these studies were used selectively in rare cases when additional postoperative evaluation was necessary based on clinical or radiographic concern. After discharge, patients obtained upright AP and lateral radiographs at routine follow-up visits at 2 weeks, 6 weeks, 3 months, 6 months, 12 months, and 24 months. Patients were excluded if their reason for operation was other than trauma (infection, tumor, or degenerative disease).

### 2.2. Outcomes Measure and Data Collection

Patients were classified by the presence of revision surgery. Revision surgery was defined as either a revision of previous instrumentation or a supplementary procedure to the index surgery. Patients who subsequently required revision surgery were identified and further analyzed to determine if the results of the radiographs obtained changed the postoperative clinical course and led to a subsequent surgical intervention. All clinical notes were evaluated for postoperative findings that could alter management, including (1) new radicular or axial neck pain, (2) new or worsening neurologic deficits, including weakness/paresis, paresthesia, or gait dysfunction, (3) dysphagia or dysphonia, (4) infection-related findings, including surgical site infection, wound complications, or documented systemic signs concerning for infection, and (5) mention of instrumentation-related complication, including fracture, loosening, or pseudarthrosis. Medical records were also reviewed to obtain patient demographic data, length of stay, mechanism of injury, surgical characteristics, spinal fracture level, and Charlson Comorbidity Index scores (CCI). Patient and trauma characteristics were evaluated between patients with and without revision surgery.

### 2.3. Statistical Analysis

All data was collected and audited in Microsoft Excel (Microsoft Office Professional Plus; Microsoft Corporation, Redmond, WA, USA). JMP Pro (Version 13.0.0 1987–2007; SAS Institute INC., Cary, NC, USA) was used for descriptive statistics. Significance level was set at *p* < 0.05. Continuous variables were tested for normality with the Shapiro-Wilk test. Unpaired *t*-tests were used for all normally distributed continuous variables and Wilcoxon sum-rank test were used for nonparametric variables. For nominal variables chi-square analysis or Fisher’s exact test were used to compare frequencies. The overall specificity, sensitivity, positive predictive value, and negative predictive value of a change in the postoperative radiograph leading to revision surgery were determined.

## 3. Results

After application of the eligibility criteria, a total of 295 patients were included in the study. The mean age was 57.3 years (range, 18–90 years), and 177 patients (60.0%) were male. Groups were similar in terms of age, sex, BMI, length of stay, and CCI score (Table 1). The majority of patients had C2-odontoid fracture (35.3%), followed by sub-axial (C3–C7) cervical spine fractures (29.8%). As for surgical procedures, 122 patients (41.4%) underwent C1–2 posterior spinal fusion (PSF), 79 (26.8%) had an anterior cervical discectomy and fusion (ACDF), and 72 (24.4%) had a subaxial PSF (Table 2).

Eleven patients (3.7%) underwent revision surgery; however, only 3 patients (1%) had abnormal findings in postoperative radiographs that prompted a change in clinical course, which ultimately led to a reoperation. No inpatient postoperative radiograph prompted a change in clinical course. All 11 patients who were reoperated presented with clinical symptoms of concern, such as neck and radicular pain, upper extremity paresthesia, or wound complications. Seven patients on the revision surgery group underwent sub-axial PSF as index surgery, demonstrating a significant difference compared to other procedures (*p* = 0.006). However, only 1 of the 7 patients had abnormal findings on postoperative radiographs; the rest had changes in clinical symptoms and normal radiographic findings. The mean days to revision surgery was 69, with a 95% confidence interval of 3–123 days. A complete description of injury pattern, index procedure, postoperative radiograph findings, and reason for revision surgery was determined for the 11 patients who were reoperated (Table 3).

**Figure 1:**
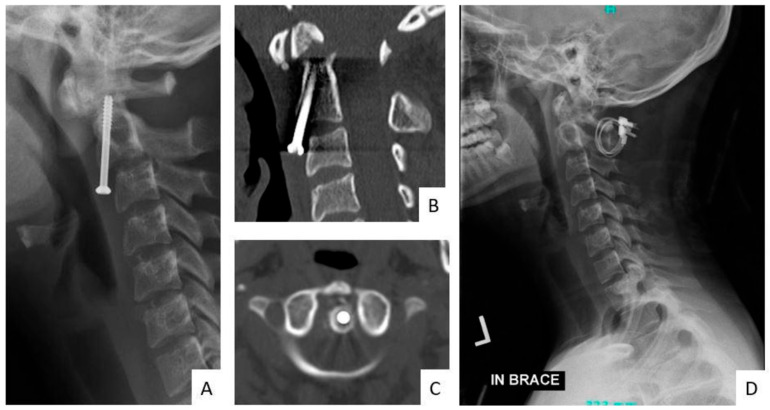
Representative case of delayed implant loosening and revision surgery. Case of an 18-year-old female with a C2 odontoid fracture was treated with odontoid screw fixation. Final intraoperative fluoroscopic images demonstrated acceptable alignment and implant positioning at the index procedure. At approximately 12 weeks postoperatively, follow-up radiographs demonstrated signs of hardware loosening/nonunion (**A**). CT was subsequently obtained for further characterization (**B**,**C**), and the patient ultimately underwent posterior C1–C2 fusion revision surgery (**D**).

Of the 3 patients that underwent revision surgery due to instrumentation failure seen in postoperative radiographs, only 1 patient had an acute instrumentation failure and dislocation at 13 days postoperatively. The patient, while in rehab, developed weakness and gait dysfunction, for which upright radiographs were obtained as part of the evaluation, showing caudal anterior screw pullout and instrumentation failure. Ultimately, CT was obtained for further evaluation, and the patient underwent combined anterior and posterior revision surgery. The other two patients also had changes in their clinical examination and eventually underwent revision surgery after 12 weeks from their initial procedure. One patient had an odontoid screw with radiographic signs of loosening/nonunion on follow-up imaging (Figure 1A); CT was subsequently obtained for further characterization (Figure 1B,C), and the patient ultimately underwent C1–C2 posterior spinal fusion (Figure 1D). The last patient, on radiographic follow-up, showed a displaced C2 nonunion fracture above previous instrumentation from C3 to T2, for which the patient underwent revision surgery extending the construct to the C1 level. Of the 8 patients with abnormal clinical findings and normal postoperative radiographs, 2 underwent revision surgery due to epidural hematomas and 6 for surgical site infection.

Sensitivity and specificity analysis showed postoperative radiographs to have a sensitivity of 27% and specificity of 100% (Table 4). The positive predictive value and negative predictive value were 100% and 97.26%, respectively.

## 4. Discussion

Obtaining postoperative radiographs before discharge and subsequently during routine follow-up visits remains a common practice after spine instrumentation. However, despite prior studies indicating limited additional clinical utility, radiographs continue to be utilized during postoperative management, particularly to document alignment, assess construct integrity, establish a postoperative baseline, and detect early instrumentation-related complications [7,13,14]. The possibility of neurologic injury as a sequela of instrumentation failure often prompts routine imaging even in clinically stable patients [2]. Nevertheless, prior literature suggests that the actual management yield of these studies is low. In one cervical fusion series evaluating 972 postoperative clinic encounters, abnormal radiographs alone changed treatment in only 6 visits [8]. Similarly, among patients with a normal postoperative history and physical examination after cervical fusion, further clinical action was taken at only 5 of 879 visits (0.57%) despite radiographic assessment [9]. In a trauma-specific thoracolumbar cohort, the in-hospital revision rate was 1.3% (6/463), and worsening neurologic status prompted reoperation more often than routine postoperative radiographs alone; notably, the sensitivity of routine inpatient radiographs was only 33.3% [10]. These data support our finding that routine postoperative radiographs after cervical spine trauma, in the absence of concerning clinical findings, have limited standalone utility in influencing postoperative management or predicting the need for revision surgery.

In our cohort, routine postoperative radiographs did not identify abnormalities suggesting the need for revision surgery in 99% of the overall study population and failed to identify such abnormalities in 73% of patients who ultimately underwent revision surgery. Instrumentation failure was identified on follow-up radiographs in only 3 patients, and notably, no abnormalities were detected on any in-hospital postoperative radiographs, including in patients who later demonstrated hardware-related failure. Importantly, all patients who underwent revision surgery had concurrent changes in clinical status or new symptoms that prompted further evaluation. Among the 8 patients with abnormal clinical findings but normal postoperative radiographs, 2 underwent revision surgery for epidural hematoma and 6 for surgical site infection. Taken together, these findings suggest that postoperative radiographs alone have limited sensitivity for detecting patients who will ultimately require revision surgery, whereas changes in neurologic examination, pain, wound status, or overall clinical course appear to be more informative in guiding further workup and management. This pattern supports a symptom-driven approach to additional imaging rather than reliance on routine radiographs as a standalone surveillance tool.

These findings are consistent with prior trauma and elective spine literature showing that postoperative clinical findings are more informative than routine radiographs when determining the need for further workup or revision surgery. In a trauma-specific thoracolumbar cohort, only 2 of 6 patients who underwent revision surgery had radiographic changes that directly prompted reoperation, whereas the remaining patients had normal postoperative radiographs but developed concerning changes in physical examination [10]. Similarly, in a cervical fusion series, only 6 of 665 postoperative encounters (0.9%) with a normal physical examination but abnormal radiographs resulted in a change in treatment course, and only 1 patient ultimately underwent revision surgery [8]. In contrast, 71 of 301 symptomatic postoperative encounters (23.1%) were associated with a change in clinical course regardless of radiographic findings, and subgroup analysis demonstrated no significant difference in radiographic utility between traumatic and degenerative pathologies [8]. At the same time, routine imaging may still occasionally identify asymptomatic hardware-related abnormalities, although prior work suggests these findings infrequently alter management in otherwise clinically stable patients [12]. When additional evaluation is needed, plain radiographs remain useful for baseline and interval follow-up, whereas CT is generally more informative for hardware integrity, loosening, and fusion/nonunion, and dynamic flexion-extension radiographs may be more useful later in follow-up when pseudarthrosis or occult instability is suspected [15,16,17]. Taken together, these studies support our findings that routine postoperative radiographs provide minimal additional value in asymptomatic patients and that evolving symptoms or examination changes remain the more clinically meaningful triggers for further evaluation after cervical spine instrumentation.

Taken together, these findings support an individualized postoperative imaging strategy rather than a uniform surveillance approach for all patients after cervical spine trauma surgery. Routine upright radiographs may still be useful for documenting alignment, establishing a postoperative baseline, and providing interval follow-up, but their value appears greatest when interpreted in conjunction with the patient’s symptoms, neurologic examination, wound status, and overall postoperative course. In contrast, CT may be more informative when the clinical question involves hardware integrity, progressive loss of alignment, loosening, nonunion, or equivocal findings on standard radiographs, whereas MRI is generally more useful when there is concern for compressive pathology, epidural hematoma, infection, or other soft-tissue complications [18,19]. Dynamic flexion-extension radiographs may also have a role later in follow-up when pseudarthrosis or occult instability is suspected, although they were not part of the routine surveillance pathway evaluated in this study [20,21]. This framework may be particularly relevant in patients with more severe injury patterns, complex constructs, persistent or worsening neurologic symptoms, or an atypical rehabilitation course, in whom selective advanced imaging may be warranted despite the limited standalone utility of routine radiographs in otherwise asymptomatic patients. Notably, newer cervical literature continues to support a symptom-guided approach to postoperative radiography overall, while still suggesting that selected higher-risk subgroups may merit closer radiographic follow-up [22].

Our study is the first to evaluate postoperative radiographs in a large cervical trauma patient population; however, there are still limitations. We argue that postoperative radiographs following cervical spine trauma have limited clinical utility, but larger studies are necessary to confirm our findings. Also, a systematic approach to assess radiographs for possible failure should be adopted to reduce variability between the surgeon and the radiologist. We are not advocating for the cessation of the use of postoperative radiographs, but in the absence of clinical findings, abnormal radiographic findings may not alter the clinical course. We also acknowledge that dynamic radiographic assessment, including flexion-extension views, may provide additional value in selected follow-up settings, particularly when pseudarthrosis, occult instability, or equivocal findings on standard radiographs are suspected. However, such stress radiographs were not part of the standardized surveillance pathway evaluated in this study, which focused specifically on routine upright AP and lateral radiographs after cervical spine trauma surgery. Likewise, postoperative CT may provide additional value in selected cases, particularly for further assessment of hardware integrity, loosening, nonunion, or equivocal findings on standard radiographs. However, routine CT was not standard practice at our institution and was not part of the surveillance pathway evaluated in this study. Rather, postoperative imaging should be individualized based on injury severity, construct complexity, postoperative course, rehabilitation progress, and the presence of clinical or radiographic concern. Further analysis of surgical technique and instrumentation details will provide more information in predicting which constructs are more prone to failure. Despite these limitations, this study offers substantial retrospective evidence on the utility of postoperative radiographs for a specific population of cervical spine trauma patients and adds to existing literature confirming that routine radiographs have limited clinical utility.

## 5. Conclusions

This study demonstrates that in the absence of clinical symptoms and changes in physical examination, postoperative radiographs have minimal clinical utility in predicting patient return to the operating room for the purpose of revision surgery. However, once abnormal findings are seen in postoperative radiographic evaluation, patients are likely to undergo reoperation.

## Figures and Tables

**Table 1:** Demographic Characteristics Between Revision Surgery and Non-Revision Cervical Spine Trauma Patients.

Characteristic, n (%)	All	Revision	Non-Revision	*p*-Value
**Patients**	295	11 (3.7)	284 (96.3)	
**Age (years), mean ± SD**	57.3 ± 20.0	56.6 ± 19.3	57.6 ± 20.0	0.86
**Sex (males)**	177 (60.0)	8 (2.74)	166 (56.85)	0.53
**BMI (kg/m^2^), mean ± SD**	26.4 ± 6.1	24.6 ± 4.8	26.4 ± 6.1	0.49
**LOS**	7.6 ± 9.9	10.8 ± 11.4	7.5 ± 9.8	0.1
**Smoking Status**				
Never	94 (52.2)	7 (3.89)	87 (48.3)	0.04
Former	39 (21.7)	1 (0.56)	38 (21.1)	-
Active	47 (26.1)	-	47 (26.1)	0.22
**Drug (active user)**	32 (17.8)	-	32 (17.8)	0.018 *
**CCI**	3.2 ± 2.6	3.1 ± 2.4	3.2 ± 2.6	0.93

* Indicates a statistically significant *p*-value < 0.05. BMI, body mass index; LOS, length of stay; CCI, Charlson Comorbidity Index.

**Table 2:** Trauma and Surgical Characteristics Between Revision and Non-Revision Surgical Cervical Spine Trauma Patients.

Characteristic, n (%)	All	Revision	Non-Revision	*p*-Value
**Patients**	295	11 (3.7)	284 (96.3)	
**Fracture Level**				0.26
**C3–C7 (Sub-axial cervical spine)**	151 (51.2)	5 (45.5)	146 (51.4)	
**C2**	112 (38.0) (percent (10.8))	3 (27.3)	109 (38.4)	
**C1–C2 (Axial cervical spine)**	32 (10.8)	3(27.3)	29 (10.2)	
**Fracture Morphology**				0.42
**Odontoid**	104 (35.3)	3 (27.3)	101 (35.6)	
**Sub-axial Cervical Spine Fracture**	79 (26.8)	4 (36.4)	73 (25.7)	
**Sub-axial Facet Dislocation/Fracture**	65 (22.1)	2 (18.1)	62 (21.8)	
**Hangman’s Fracture ‡**	10 (3.4)	-	10 (3.5)	
**Axial Lateral Mass Fracture**	10 (3.4)	-	10 (3.5)	
**Other**	10 (3.4)	2 (18.1)	8 (2.8)	
**Mechanism of Injury**				
**Motor Vehicle Accident**	124 (44.8)	6 (54.6)	118 (41.5)	0.54
**Fall**	102 (34.5)	2 (18.2)	100 (35.2)	0.34
**Other**	47 (15.9)	3 (27.3)	44 (15.5)	0.39
**Surgical details**				
**Instrumented Segments, mean ± SD**	1.37 ± 0.734	1.33 ± 0.51	1.36 ± 0.75	0.74
**Procedure †**				0.019 *
**C1–2 PSF**	122 (41.4)	1 (9.1)	121 (42.6)	0.03
**ACDF**	79 (26.8)	1 (9.1)	78 (27.5)	0.3
**Sub-axial PSF**	72 (24.4)	7 (63.6)	65 (22.9)	0.006 ***
**360 Fusion**	10 (3.8)	1 (9.1)	9 (3.2)	0.32
**Odontoid Screw**	6 (2.0)	1 (9.1)	5 (4.5)	0.21
**ACCF**	3 (1.0)	-	3 (1.1)	-

‡ Refers to Traumatic Spondylolisthesis of Axis (C1) * Indicates a statistically significant *p*-value < 0.05. *** indicates a statistically significant *p*-value < 0.008 † Post hoc analysis done with multiple Fisher’s exact tests and statistical significance assessed after alpha corrected with Bonferroni correction ((α = 0.05)/6 = 0.008). PSF, posterior spinal fusion; ACDF, anterior cervical discectomy and fusion; ACCF, anterior cervical corpectomy and fusion.

**Table 3:** Description of Initial Trauma and Reason for Revision Surgery Cases After Cervical Spine Trauma.

Age/Sex	Days D/C to Rev	MOI	Injury Pattern	Index Procedure	Postoperative Clinical Findings	Postoperative Radiograph Findings	Reason for Revision Surgery	Procedure
**18 F**	89	MVA	C2 odontoid fracture	Odontoid screw	Suboccipital pain, neurologically intact	Surgical hardware along C1 and C2, fracture non-union	Instrumentation failure	C1–2 posterior spinal fusion
**31 M**	201	MVA	C6–7 facet dislocation	C3–4 ACDF and C6–7 PSF	External posterior wound dehiscence and failure to heal with local wound care		Surgical Site Infection	Irrigation and debridement with muscle flap advancement
**50 M**	17	MVA	C7–T1 fracture-dislocation	C6–T1 PSF	Grossly infected wound with purulent drainage	Persistent widening of C6-T1 was observed	Surgical Site Infection	Irrigation and debridement
**84 M**	3	Fall	C5–6 extension-distraction	C3–T1 PSF	Readmitted from rehab for urosepsis, altered mental status, and worsening lower extremity weakness	X-ray: No signs of instrumentation loosening. MRI: revealed epidural hematoma	Epidural hematoma	C6–7 and T1–3 Laminectomy, removal of C7 instrumentation, and extension of construct from C7–T3.
**77 M**	301	Fall	C6–7 dislocation	C4–T4 PSF	External wound dehiscence and failure to heal with local wound care	Vertebral levels are obscured by overlapping density	Surgical Site Infection	Removal of hardware with irrigation and debridement
**60 M**	39	MVA	C6–7 flexion—distraction	C6–T3 PSF	Wound dehiscence and purulent drainage	No signs of loosening in instrumentation.	Surgical Site Infection	Irrigation and debridement
**55 F**	23	Horse-riding	C2 odontoid fracture	C1–2 PSF	Chills, pain, wound dehiscence and purulent drainage	No signs of loosening in instrumentation.	Surgical Site Infection	Irrigation and debridement
**57 M**	13	MVA	C6–7 facet dislocation	C6–7 ACDF	Increasing pain, weakness, and gait dysfunction	Anteriorly displaced C7 screws, hardware failure, and dislocation	Instrumentation failure	Revision fusion C6–7 with ACDF and posterior instrumentation C6–7
**73 M**	112	MVA	C2 odontoid fracture	C3–T2 PSF	Increasing pain at the cranio-cervical junction, exacerbated by any range of motion	X-ray: right-sided dislocation of non-union odontoid fracture MRI showed Displaced C2 fracture above instrumentation	Instrumentation failure	Removal of instrumentation at C3 and extension of construct to C1
**59 F**	56	Fall	C7 pedicle fracture	C6–T1 PSF	Wound dehiscence and purulent drainage	No signs of loosening in instrumentation.	Surgical Site Infection	Irrigation and debridement
**60 M**	7	Fall	C7 pars fracture	C6–T1 PSF	Postoperative urinary retention and worsening lower extremity weakness	X-ray: No signs of loosening of instrumentation MRI: revealed hematoma over C7 causing cord compression	Epidural hematoma	C7 laminectomy, hematoma drainage, and re-instrumentation and insertion of T1 pedicle screw

MOI, Mechanisms of Injury; PSF, posterior spinal fusion; ACDF, anterior cervical discectomy and fusion.

**Table 4:** Sensitivity, Specificity, And Positive and Negative Predictive Value of Cervical Spine Postoperative Radiographs.

	Revision Surgery	No Revision Surgery	
**Radiographic Changes**	3 (TP)	0 (FP)	PPV: 100%
**No Radiographic Changes**	8 (FN)	284 (TN)	NPV: 97.26%
	Sensitivity: 27%	Specificity: 100%	

TP, true positive; FP, false positive; PPV, positive predictive value; FN, false negative; TN, true negative; NPV, negative predictive value.

## Data Availability

Data are available upon request from the authors.

## Abbreviations

The following abbreviations are used in this manuscript:

IRB	Insitutional Review Board
ACDF	Anterior Cervical Diskectomy and Fusion
PSF	Posterior Spinal Fusion
BMI	Body Mass Index
CCI	Charleson Comorbidity Index
CT	Computerized Topography
ODI	Oswestry Disability Index

## References

[1] Milby A.H., Halpern C.H., Guo W., Stein S.C. (2008). Prevalence of cervical spinal injury in trauma. Neurosurg. Focus.

[2] Torretti J.A., Sengupta D.K. (2007). Cervical spine trauma. Indian J. Orthop..

[3] Zileli M., Osorio-Fonseca E., Konovalov N., Cardenas-Jalabe C., Kaprovoy S., Mlyavykh S., Pogosyan A. (2020). Early Management of Cervical Spine Trauma: WFNS Spine Committee Recommendations. Neurospine.

[4] Fredø H.L., Rizvi S.A.M., Lied B., Rønning P., Helseth E. (2012). The epidemiology of traumatic cervical spine fractures: A prospective population study from Norway. Scand. J. Trauma Resusc. Emerg. Med..

[5] Goldberg W., Mueller C., Panacek E., Tigges S., Hoffman J.R., Mower W.R., NEXUS Group (2001). Distribution and patterns of blunt traumatic cervical spine injury. Ann. Emerg. Med..

[6] Kwon B.K., Fisher C.G., Boyd M.C., Cobb J., Jebson H., Noonan V., Wing P., Dvorak M.F. (2007). A prospective randomized controlled trial of anterior compared with posterior stabilization for unilateral facet injuries of the cervical spine. J. Neurosurg. Spine.

[7] Bartels R.H.M.A., Beems T., Schutte P.J., Verbeek A.L.M. (2010). The rationale of postoperative radiographs after cervical anterior discectomy with stand-alone cage for radicular pain. J. Neurosurg. Spine.

[8] Shau D.N., Bible J.E., Samade R., Gadomski S.P., Mushtaq B., Wallace A., McGirt M.J., O’Neill K.R., Devin C.J. (2012). Utility of postoperative radiographs for cervical spine fusion: A comprehensive evaluation of operative technique, surgical indication, and duration since surgery. Spine.

[9] Grimm B.D., Leas D.P., Glaser J.A. (2013). The utility of routine postoperative radiographs after cervical spine fusion. Spine J..

[10] Pyun J., Camacho J.E., Usmani M.F., Weir T.B., Yousaf O., Sood A., Vishwanath V., Jolissaint J., Shasti M., Koh E.Y. (2019). The Utility of In-Hospital Postoperative Radiographs Following Surgical Treatment of Traumatic Thoracolumbar Injuries. Clin. Spine Surg..

[11] Shin H.-K., Jeong H.-J., Kim E., Park J.H., Park S.-J., Cho Y. (2017). Should We Check the Routine Postoperative MRI for Hematoma in Spinal Decompression Surgery?. Clin. Orthop. Surg..

[12] Desai A., Pendharkar A.V., Swienckowski J.G., Ball P.A., Lollis S., Simmons N.E. (2015). Utility of Routine Outpatient Cervical Spine Imaging Following Anterior Cervical Corpectomy and Fusion. Cureus.

[13] Ugokwe K.T., Kalfas I.H., Mroz T.E., Steinmetz M.P. (2008). A review of the utility of obtaining repeated postoperative radiographs following single-level anterior cervical decompression, fusion, and plate placement. J. Neurosurg. Spine.

[14] Molinari R.W., Hunter J.G., McAssey R.W. (2012). In-hospital postoperative radiographs for instrumented single-level degenerative spinal fusions: Utility after intraoperative fluoroscopy. Spine J..

[15] Gruskay J.A., Webb M.L., Grauer J.N. (2014). Methods of evaluating lumbar and cervical fusion. Spine J..

[16] Nouh M.R. (2012). Spinal fusion-hardware construct: Basic concepts and imaging review. World J. Radiol..

[17] Lambrechts M.J., D’Antonio N.D., Karamian B.A., Toci G.R., Sherman M., Canseco J.A., Kepler C.K., Vaccaro A.R., Hilibrand A.S., Schroeder G.D. (2022). What is the role of dynamic cervical spine radiographs in predicting pseudarthrosis revision following anterior cervical discectomy and fusion?. Spine J..

[18] Derakhshan A., Lubelski D., Steinmetz M.P., Benzel E.C., Mroz T.E. (2015). Utility of Computed Tomography following Anterior Cervical Diskectomy and Fusion. Glob. Spine J..

[19] Corona-Cedillo R., Saavedra-Navarrete M.-T., Espinoza-Garcia J.-J., Mendoza-Aguilar A.-N., Ternovoy S.K., Roldan-Valadez E. (2021). Imaging Assessment of the Postoperative Spine: An Updated Pictorial Review of Selected Complications. Biomed. Res. Int..

[20] Lin W., Ha A., Boddapati V., Yuan W., Riew K.D. (2018). Diagnosing Pseudoarthrosis After Anterior Cervical Discectomy and Fusion. Neurospine.

[21] Leven D., Cho S.K. (2016). Pseudarthrosis of the Cervical Spine: Risk Factors, Diagnosis and Management. Asian Spine J..

[22] Finneran M.M., Nardone E.M. (2025). Role of routine radiographs after anterior cervical discectomy and fusion. Neurosurg. Rev..

